# Efficacy and Safety of Topical Application of Olive Oil for Preventing Pressure Ulcers: A Systematic Review and Meta-Analysis of Randomized Controlled Trials

**DOI:** 10.3390/ijerph192214921

**Published:** 2022-11-13

**Authors:** Akram Hernández-Vásquez, Fabriccio J. Visconti-Lopez, Cielo Cabanillas-Ramirez, Deysi Díaz-Seijas, Jessica Meléndez-Escalante, Daniel Comandé, Marilina Santero

**Affiliations:** 1 Centro de Excelencia en Investigaciones Económicas y Sociales en Salud, Vicerrectorado de Investigación, Universidad San Ignacio de Loyola, Lima 15024, Peru; 2 Facultad de Ciencias de la Salud, Universidad Peruana de Ciencias Aplicadas, Lima 15023, Peru; 3 Instituto Nacional Cardiovascular INCOR, EsSalud, Lima 15072, Peru; 4 Instituto de Efectividad Clínica y Sanitaria (IECS-CONICET), Buenos Aires C1414CPV, Argentina; 5 Iberoamerican Cochrane Centre, IIB Sant Pau—Servei d’Epidemiologia Clínica i Salut Pública, Hospital de la Santa Creu i Sant Pau, 08041 Barcelona, Spain

**Keywords:** humans, pressure ulcer, bedsore, olive oil, prevention and control, GRADE approach, randomized controlled trial

## Abstract

The purpose was to identify and summarize the existing evidence on the efficacy and safety of the topical application of olive oil for preventing pressure ulcers (PUs). We included only randomized controlled trials (RCTs) involving patients at risk of developing PUs, testing the topical application of olive oil versus other products for PU prevention. We assessed the risk of bias using the RoB 2 tool, and the certainty of the evidence with GRADE. Four RCTs met the eligibility criteria. All studies were judged at a low risk of bias overall. The meta-analysis showed that the clinical efficacy of olive oil for prevention occurs by reducing the incidence of PUs (RR = 0.56, 95% CI = 0.30 to 0.79, *I*^2^ = 0%); with no differences in adverse effects, it may be associated with a shorter development time of PUs and shorter hospital stays. The certainty of the evidence assessed by the GRADE approach was moderate and low. The topical application of olive oil is effective and safe in reducing the incidence of PUs compared to other treatments. These findings could provide new insights into olive oil as a preventive and alternative treatment for PUs as it is accessible and inexpensive compared to other products.

## 1. Introduction

Pressure injuries, commonly referred to as pressure ulcers (PUs), are a common complication for people who are forced to lay in bed for long periods of time. and increase morbidity and mortality in people with chronic conditions [1,2]. Their reported prevalence is 26.6% and 16.2% in the hospital and intensive care unit (ICU) context, respectively [3]. Moreover, the high costs of treating PUs represent a considerable burden for health systems. Total costs have been estimated annually at GBP 2.1 billion in the United Kingdom [4], and USD 26.8 billion in the United States [5].

People at a high risk of developing PUs require early prevention measures [6]. Currently, besides cleaning, repositioning, early mobilization, and using support surfaces such as pillows, skin moisturizing is highly recommended [2]. Therefore, the topical application of emollients, moisturizers such as hyperoxygenated fatty acids (HOFA), and silicone creams is suggested [2].

An interesting alternative for PU prevention is olive oil. It contains essential fatty acids, such as linoleic and oleic acid, which improve hydration and cause a protective, emollient, and regenerating effect on the skin [7]. The adverse events that can develop due to the use of olive oil or fatty acids against pressure ulcers are related to local adverse effects, such as rash, itching, stinging, or pain [8]. In 2018, a systematic review evaluated the efficacy and safety of the prevention of Pus, including data from two studies; however, the authors obtained inconclusive results about the incidence of PUs between groups [8]. Since then, new randomized controlled trials (RCTs) have been completed that provide more information about the role of olive oil in the prevention of PUs.

Therefore, it is vital to understand the potentialities and uses of olive oil in order to improve current prevention strategies. Thus, this study aims to identify and summarize existing evidence on the efficacy and safety of the topical application of olive oil for preventing PUs.

## 2. Materials and Methods

### 2.1. Study Design

This systematic review (SR) and meta-analysis follows the Preferred Reporting Items for Systematic Review and Meta-Analysis (PRISMA) reporting guidelines [9]. The protocol was previously registered in the Prospective International Registry of Systematic Reviews (PROSPERO) database (registration number CRD42022297918). Moreover, all materials and data are available in the Supplementary Materials to facilitate the reproducibility and transparency of this review.

### 2.2. Data Sources and Search Strategy

We searched for all potentially relevant studies published from inception until 14 December 2021. A systematic literature search was conducted using eight electronic bibliographic databases: PubMed, EMBASE (access via OVID), Cochrane Library, CINAHL, Web of Science, Scopus, Global Health, and LILACS. The search strategy was carried out by an experienced medical research librarian (DC) and validated by the research team. Search terms focused on these keywords: bedsore, pressure ulcer, pressure sore, decubitus ulcer, and olive oil (see Supplementary Table S1). In addition to the electronic search, reference lists of all included studies were inspected for further relevant studies.

We imported records obtained from all databases into the EndNote X9 reference management software to eliminate duplicate publications, following the procedures described by Bramer et al. [10].

### 2.3. Identification and Selection of Studies

We used the PICOS framework (Patients, Intervention, Comparison, Outcomes, Study design) to guide our eligibility criteria [11].

We included only RCTs meeting all the following inclusion criteria: (a) investigate patients at risk of developing PUs, in any care setting; (b) assess topical application of olive oil as the intervention for PU prevention; (c) compare other products for PU prevention. Studies that do not explicitly define the control group, or studies with a placebo as the control group, were also included. We excluded studies meeting any of the following criteria: (a) include hospitalized patients with pre-existing PUs; (b) published in a language other than English, Spanish, or Portuguese; (c) other types of publications such as review articles, case reports, book chapters, editorials, and letters.

Two previously trained reviewers (FJVL and CBR) independently screened the titles and abstracts for relevant articles prior to appraising the full texts. A third reviewer (AHV) solved any disagreements. If necessary, the reviewers contacted the principal authors of the identified studies via email for details, but if no response was received within 2 weeks, the study was excluded. For all the selection processes, we used Rayyan, a free web app that helps expedite the initial screening of abstracts and titles using a process of semi-automation while incorporating a high level of usability [12].

### 2.4. Outcomes

The primary outcome was the incidence of PUs, defined as the number or proportion of patients developing a PU on any part of the body that is grade 1 (nonblanchable erythema), 2 (partial skin loss), 3 (full-thickness skin loss), or 4 (deep-tissue destruction) during their stay in the care setting [13]. Secondary outcomes included: (a) time to ulcer development (as a time-to-event outcome); (b) adverse events (measured as moderate or severe adverse events, according to the standardized classification); (c) quality of life (measured with validated scales); and (d) length of hospital stay (measured as the total days of admission during the follow-up period).

### 2.5. Data Extraction

Two reviewers (FJVL and CCR) extracted data independently from all included studies, using a predefined form previously piloted. A second reviewer (AHV) cross-checked this process. The study authors were contacted for missing data or clarification where appropriate. The types of data extracted included: first author, year of publication, country of study, language of publication, study design, setting, type of participants, type of intervention, control, outcomes, duration and follow-up, sample size, population characteristics, and funding/conflict of interest.

### 2.6. Quality Assessment

Two reviewers (FJVL and CCR) assessed the risk of bias of each included RCT using the Cochrane Risk of Bias tool (RoB 2). A third reviewer (MS) cross-checked this assessment. RoB 2 is structured into a fixed set of domains of bias, focusing on different aspects of trial design, conduct, and reporting. We considered six domains for the assessment: (i) risk of bias arising from the randomization process, (ii) risk of bias due to deviations from the intended interventions, (iii) missing outcome data, (iv) risk of bias in the measurement of the outcome, (v) risk of bias in the selection of the reported result, and (vi) overall risk of bias. Within each domain, a series of questions (“signalling questions”) aim to elicit information about features of the trial that are relevant to the risk of bias. A proposed judgement about the risk of bias arising from each domain is generated by an algorithm, based on answers to the signaling questions. The judgement can be a “Low” or “High” risk of bias, or can express “Some concerns”.

### 2.7. Statistical Analysis

For dichotomous outcomes, we present a risk ratio (RR) with the 95% confidence interval (95% CI). When it was allowed, we performed a meta-analysis using random-effects models (inverse variance and Mantel–Haenszel method) to calculate a weighted summary effect estimate RR and a 95% CI. Meta-analyses were performed using the meta package in the R programming language and RStudio [14]. Results were graphically represented using forest plot graphs. The analysis codes are available in the Supplementary Materials.

We assessed heterogeneity using the *I*^2^ statistics, and we considered that heterogeneity might not be important when *I*^2^ < 40% [15]. Tau^2^ defined the between-studies variance. Publication bias was not assessed due to the number of studies pooled for each meta-analysis being less than ten [15]. Statistical significance was defined as a *p*-value < 0.05.

We performed a sensitivity analysis, excluding one study at a time to verify the stability of the results and sources of heterogeneity, by using the leave-one-out method; to assess the influence of small-study effects on the results of our meta-analysis, the fixed-effects and random-effects estimates of the intervention effect were compared.

### 2.8. Certainty of the Evidence

We assessed the certainty of the evidence for each outcome according to the Grading of Recommendations Assessment, Development, and Evaluation (GRADE) guidance [16], and made a “Summary of Findings” (SoF) table. We classified the certainty of the evidence for each outcome as “high”, “moderate”, “low”, or “very low”. Given that the data come from RCTs, we initially rated their certainty as high, which was downgraded in the presence of an important bias, indirectness or inconsistency in results, imprecision in estimates, or suspicion of publication bias.

### 2.9. Ethics

Our study did not require institutional ethics approval because it was an analysis of publicly accessible aggregated secondary data. We did not collect deeply personal, sensitive, or confidential information from participants.

## 3. Results

### 3.1. Study Selection

**Figure 1:**
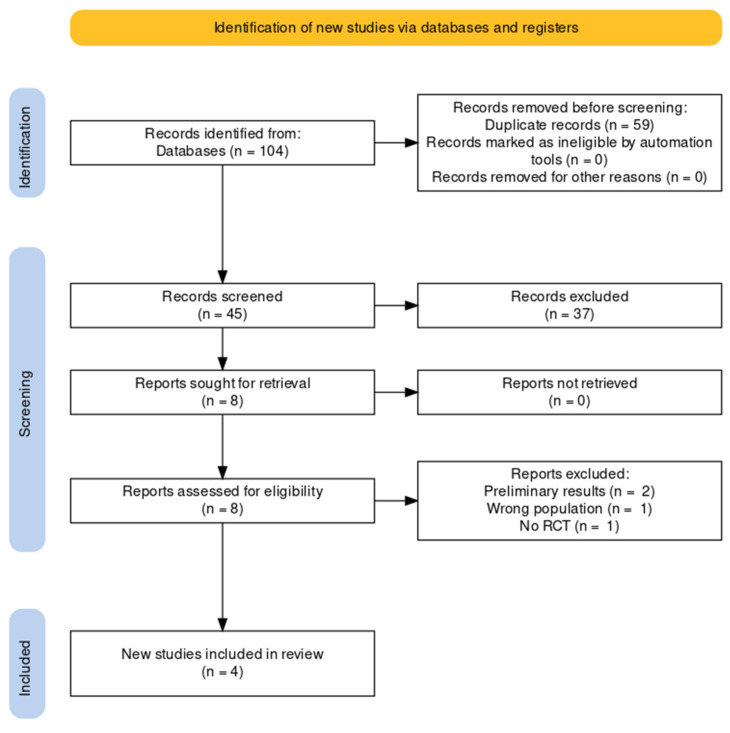
PRISMA flow diagram.

Our initial systematic literature search identified 104 records. First, we excluded duplicate records, leaving a total of 45 articles. Second, we excluded 37 records (reasons are presented in the Supplementary Materials). After removing duplicates and performing the title and abstracts screening (phase 1), eight records remained for full-text reading (phase 2). According to the eligibility criteria, four studies were excluded (Supplementary Table S3), and four RCTs were, finally, included for qualitative and quantitative analysis [17,18,19,20]. Supplementary Table S3 presents all excluded references and reasons for exclusion [21,22,23,24]. Figure 1 presents an overview of the selection process.

### 3.2. Study Characteristics

The four studies on patients at risk of developing PUs accounted for a total of 1601 subjects (820 intervention and 781 control subjects) (1079 women; 522 men; female to-male ratio 2:1) with a mean age ranging between 17.3 [20] and 84.4 [17] years. The studies were conducted in three different countries, Spain, Iran, and Turkey, and sample sizes ranged from 70 [20] to 831 subjects [18]. Table 1 summarizes the general characteristics of the four included RCTs, all published between 2015 and 2020. Two out of the four studies included patients from intensive care units. The topical application of olive oil in any form of presentation was evaluated against several different active comparators (e.g., HOFA) or a placebo. All the studies reported incidence of PUs. Three studies reported adverse events [17,18,19].

One study reported the time to ulcer development [19], and one study reported the length of hospital stay [20]. None of the RCTs reported on quality of life.

### 3.3. RoB 2 within and across Studies

**Figure 2:**
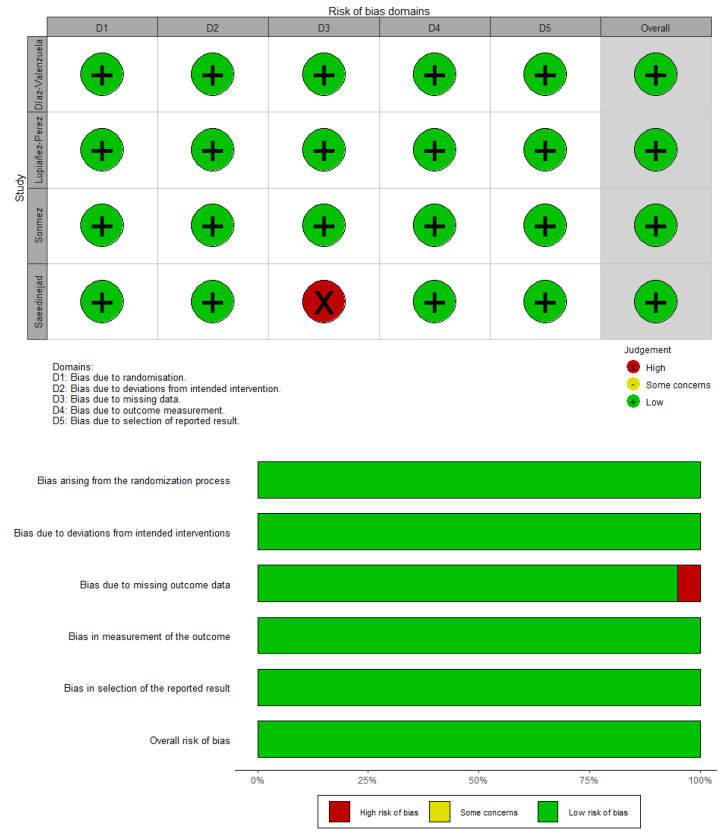
Risk of bias summary, assessed by RoB 2: authors’ judgments for each included study.

All studies were judged to have a low risk of bias overall [17,18,19,20]. One study reported substantial missing data [20]. Further information about the risk of bias assessment can be found in Figure 2.

### 3.4. Incidence of Pressure Ulcers

**Figure 3:**
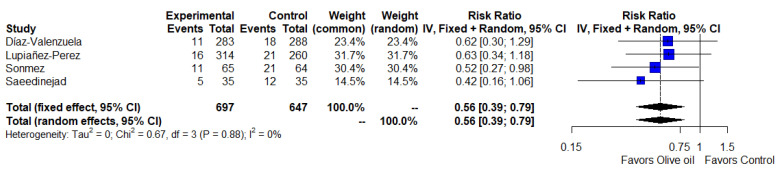
Efficacy analysis (forest plot). The blue squares represent the point estimate of the effect size and sample size. The lines on either side of the box represent a 95% confidence interval (CI). The rhombus represents pooled results [17,18,19,20].

Figure 3 provides a meta-analysis of the four studies showing that the clinical efficacy of olive oil was better than the control (RR = 0.56, 95% CI = 0.39 to 0.79, *I*^2^ = 0%) [17,19,20,21]. Additionally, the analysis of data for the subgroup of patients in the ICU showed an effect of olive oil in reducing the incidence of PUs (RR = 0.48, 95% CI = 0.28 to 0.82, *I*^2^ = 0%) and no difference in studies with HOFAs (RR = 0.63, 95% CI = 0.39 to 1.01, *I*^2^ = 0%) as the control (Figures S1 and S2).

### 3.5. Adverse Events

**Figure 4:**
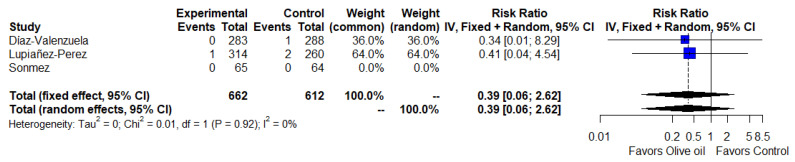
Safety analysis (forest plot). The blue squares represent the point estimate of the effect size and sample size. The lines on either side of the box represent a 95% confidence interval (CI). The rhombus represents pooled results [17,18,19].

Figure 4 provides a meta-analysis of two studies showing no difference in the association of olive oil with adverse events (RR = 0.39, 95% CI = 0.06 to 2.62, *I*^2^ = 0%) [17,18].

### 3.6. Time to Ulcer Development

Only one study including 129 participants in the ICU suggested that olive oil may be associated with a longer number of days for PU development (olive oil: 10.45 +/− 5.20 days vs. control: 7.50 +/− 5.43) [19].

### 3.7. Hospital Stay

Only one study with 70 participants in the ICU suggested that olive oil may be associated with an improvement in the average length of stay in days (olive oil: 23.25 ± 28.70 vs. control: 25.93 ± 30.63) [20].

### 3.8. Sensitivity Analysis

**Figure 5:**
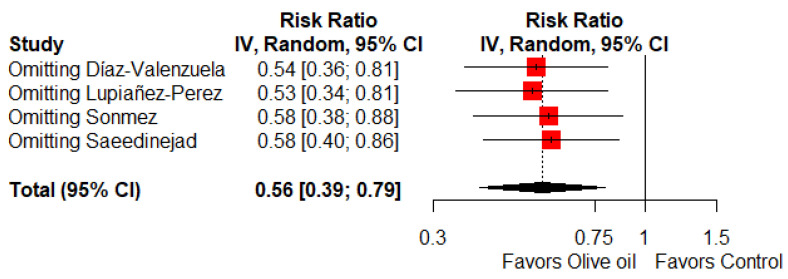
Sensitivity analysis (forest plot). The red squares represent the point estimate of the effect size and sample size. The lines on either side of the box represent a 95% confidence interval (CI). The rhombus represents pooled results [17,18,19,20].

We performed sensitivity analyses based on the leave-one-out method, and the obtained results were consistent with the main analysis for the primary outcome (Figure 5).

### 3.9. Certainty of Evidence

Table 2 presents the certainty assessment for each primary outcome according to the GRADE approach [25,26]. The evidence regarding efficacy is of moderate certainty, meaning that further research is likely to have an important impact on our confidence in the estimate of effect and may change the estimate. The evidence regarding safety is of low certainty, meaning that further research is very likely to have a significant impact on our confidence in the estimate of effect and is likely to change the estimate.

## 4. Discussion

We systematically revised and summarized the existing evidence on the effects and safety of the topical application of olive oil for preventing PUs. Of all the databases explored, there are few RCTs of good quality. After meta-analyses of the data, it seems that olive oil has an effect in reducing the incidence of PUs, is safe, and may be associated with a longer development time of PUs and shorter hospital stays. However, the certainty of the evidence assessed by the GRADE approach is still moderate and low.

The findings of our SR are directly in line with previous studies. For example, a SR from 2016, contrasting the efficacy of olive oil against hyperoxygenated fatty acids, reported that extra virgin olive oil reduces the incidence of PUs, being as effective as hyperoxygenated fatty acids in preventing these injuries [27]. Regarding safety, the authors pointed out that patients in the olive oil group did not report any adverse events. Our study reinforces their conclusions and offers a systematical review of the literature thus far. We identified a lower incidence of PUs in the olive oil group compared to the control group. In our SR, we have included four RCTs, which consider the population of Spain, Turkey, and Iran. Two out of four studies included patients in intensive care units, and all studies evaluated the effectiveness of the topical application of olive oil for PU prevention. A different SR, from 2018, compared the effect of fatty acids versus olive oil as topical agents on the development of PUs and included two RCTs in their meta-analysis [8]. The authors concluded that both interventions are safe, which is similar to our findings, as we did not find any difference in the incidence of adverse events, determining that there is no clear difference in the incidence of adverse events between fatty acids and olive oil (one trial, n = 831; RR 2.22; 95% CI 0.20 to 24.37; low-certainty evidence, downgraded for very serious imprecision).

From the results of our SR, we propose olive oil as an effective and safe preventive alternative treatment in conjunction with other measures such as risk assessment, skin care, repositioning, and well-controlled blood sugars. Although there are other preventive measures for PUs in patients at risk of developing them, olive oil is shown as an economic and frequently used option. For example, in Spain, where the studies with the largest sample size were carried out, Oleicopiel is a product manufactured and distributed nationwide by a pharmacist; thus, it can be assumed that it is a frequently used product. Likewise, from the RCT of Lupiañez-Pérez, it was concluded that olive oil as a preventive treatment allows considerable savings in direct costs, being EUR 10,192 less expensive than hyperoxygenated fatty acids [22]. In our study, we could also determine that a longer time to ulcer development and a shorter hospital stay may be associated with the application of olive oil. This leads to lower costs for the patient and the health system. It is important to highlight that numerous studies on mice have shown that the topical application of olive oil on PUs improves wound healing through the effects of anti-inflammation, a reduction of oxidative damage, and the promotion of dermal reconstruction [28,29,30,31]. Equally, a RCT showed that a mixture of olive oil, sesame oil, and honey was a useful treatment for burns by preventing infections and accelerating tissue repair [32]. A Cochrane review assessing the topical application of fatty acids as an intervention showed that compared to olive oil, data from two trials showed no clear difference in the incidence of PUs [8].

The present study also involved certain limitations. First, we do not address other regional databases. However, an extensive literature search was conducted on several scientific bibliographic databases, including PubMed, EMBASE (access via OVID), Cochrane Library, CINAHL, Web of Science, Scopus, Global Health, and LILACS. Second, not all the studies evaluated presented information about the secondary outcomes of this study, and therefore, it is not possible to determine with certainty the effect of the application of olive oil on these outcomes. Nevertheless, the study synthesizes the available evidence about the main objective of our SR: the incidence of PUs. Third, only four studies conducted in three countries, of which two were from Spain, were included in this study. Considering the characteristics of the healthcare system of this country, it becomes difficult to extrapolate the results to low- and middle-income countries. Fourth, another relevant limitation of this study is the difference between the attention of the nurses in an intensive care unit and a nursing home. The number of nurses who take care of the patient is distinct in ratio. The literature does not specify a ratio for each category, but it has been demonstrated that the nurse–patient ratio is one of the determining factors of the patient outcome [33,34]. Likewise, if there are more nurses for each patient, the quality of health attention will be better than in cases with fewer nurses per patient. Last, even though most of the included studies were double-blinded, and authors assured that they used the necessary measures for the patients, professionals, or researchers applying the treatment to not know which of the two products was used, it must be taken into account that olive oil has a particular odor and risk bias could not be avoided completely.

These findings could provide new insights into olive oil as a preventive and alternative treatment for PUs based on it being accessible and inexpensive compared to the other products available to prevent Pus, and as it is a widely available product. Although further evidence is needed, the present study contributes to a better understanding of the efficacy and safety of olive oil in the prevention of PUs.

For future research, it would be important to consider this preventive method in different settings, such as immobilized, post-operative patients, or patients with certain comorbidities [35]. In this way, we could obtain more information and better data related to the performance of olive oil in the prevention of PUs. It would also be relevant to conduct research about instructions for use of this product, such as for the timing and method of application, and to carry out studies that evaluate values and preferences when making shared decisions. The results should also be explored, considering the various types of olive oil available in the market.

## 5. Conclusions

In conclusion, the present study contributes to a better understanding of a preventive treatment little studied. We found that there is an effect of olive oil in reducing the incidence of PUs, and that it is safe. However, it should be taken into consideration that there is not much evidence and the certainty of the results is not the best. For that reason, future research could deepen our conclusions and new variables of interest could be addressed, such as patient product acceptability, adverse events, and economic assessments to help healthcare managers make rational decisions.

## Figures and Tables

**Table 1:** Characteristics of included studies, RCTs, studying olive oil for preventing pressure ulcers (*n* = 4).

Author (Year) and Country	Population	Intervention	Comparator	Outcomes	Aims
Díaz-Valenzuela, 2019 [17] Spain	Nursing home residents at risk of PU onset (Braden Scale score < 14 points) *n* = 571 (I 283; C 288)	Extra virgin olive oil solution (Oleicopiel, Potosi-10, Orcera, Jaen, Spain)	HOFA solution (Mepentol, Bama-Geve, Barcelona, Spain)	IU; adverse events	To compare the effectiveness of the topical application of olive oil versus HOFA to prevent PUs in elderly residents at risk; to compare the safety (adverse effects) of the two treatments
Lupiañez-Perez, 2015 [18] Spain	Immobilized patient programme receiving the home nursing service *n* = 831 (ITT: I 437, C 394; PP: I 314, C 260)	Liquid spray form, containing 97% extra virgin olive oil and 3% Hypericum perforatum and peppermint.	HOFA-based product	IU; adverse events	To assess the effectiveness of the use of olive oil, comparing it with hyperoxygenated fatty acids, for immobilized home-care patients at risk of suffering from PUs
Sönmez, 2020 [19] Turkey	ICU patients *n* = 129 (I 65, C 64)	Extra virgin olive oil (Taris, Memecik, Southern Aegean Region natural extra virgin olive oil)	No moisturizing product	IU; adverse events; time to ulcer development	To examine the effect of topically applied EVOO for the prevention of PU; to evaluate the development of PU according to various patient characteristics
Saeedinejad, 2017 [20] Iran	ICU patients *n* = 70 (I 35, C 35)	Olive oil	Not reported	IU; hospital stay	To determine the effects of olive oil on the prevention of PUs

HOFA: hyperoxygenated fatty acid; EVOO: extra virgin olive oil; ICU: intensive care unit; PUs: pressure ulcers; IU: incidence of ulcer; I: intervention group; C: control group; ITT: intention-to-treat; PP: per protocol.

**Table 2:** Summary of Findings (SoF) table.

**Topical application of olive oil compared to other pressure ulcer prevention products for people of any age or sex with risk of developing a pressure ulcer**					
**Patient or population:** people of any age or sex with risk of developing a pressure ulcer **Setting:** hospital, nursing homes, homes **Intervention:** topical application of olive oil **Comparison:** other pressure ulcer prevention products					
**Outcomes**	**№ of participants (studies)** **Follow-up**	**Certainty of the evidence** **(GRADE)**	**Relative effect** **(95% CI)**	**Anticipated absolute effects**	
				**Risk with other pressure ulcer prevention products**	**Risk difference with topical application of olive oil**
Incidence of pressure ulcers	1344 (4 RCTs)	⨁⨁⨁◯ Moderate^a^	**RR 0.56** (0.39 to 0.79)	111 per 1000	**49 fewer per 1000** (68 fewer to 23 fewer)
Adverse events (measured as moderate or severe adverse events, according to standardized classification) (safety)	1274 (3 RCTs)	⨁⨁◯◯ Low^a,b^	**RR 0.39** (0.06 to 2.62)	5 per 1000	**3 fewer per 1000** (5 fewer to 8 more)
*The risk in the intervention group (and its 95% confidence interval) is based on the assumed risk in the comparison group and the **relative effect** of the intervention (and its 95% CI). **CI**: confidence interval; **RCTs**: randomized controlled trial; **RR**: risk ratio					
**GRADE Working Group grades of evidence** **High certainty:** we are very confident that the true effect lies close to that of the estimate of the effect. **Moderate certainty:** we are moderately confident in the effect estimate: the true effect is likely to be close to the estimate of the effect, but there is a possibility that it is substantially different. **Low certainty:** our confidence in the effect estimate is limited: the true effect may be substantially different from the estimate of the effect. **Very low certainty:** we have very little confidence in the effect estimate: the true effect is likely to be substantially different from the estimate of effect.					

**Explanations**: a. Few initial studies are available. Early positive studies (small in size) are suspect. b. A recommendation or clinical course of action would differ if the upper versus the lower boundary of the CI represented the truth. The GRADEpro GDT software (Guideline Development Tool) was used to summarize the results [26].

## Data Availability

The data presented in this study are available in the Supplementary Materials.

## Supplementary Materials

The following supporting information can be downloaded at: https://www.mdpi.com/article/10.3390/ijerph192214921/s1, Table S1: Search strategies; Table S2: List of records identified in databases after removing duplicates; Table S3: Excluded records based on title and abstract review; Table S4: Excluded studies based on full-text review. Table S5: The PRISMA Statement checklist. Figure S1: Subgroup analysis of patients in ICU (forest plot). Figure S2: Subgroup analysis of studies with HOFA as control (forest plot). File S1: Analysis codes. File S2: RIS of records identified in databases after removing duplicates. Reference [36] is cited in the supplementary materials.
