# Supplementary material for: Efficacy and Safety of Topical Application of Olive Oil for Preventing Pressure Ulcers: A Systematic Review and Meta-Analysis of Randomized Controlled Trials
Source: Int J Environ Res Public Health. 2022 Nov 13;19(22):14921. doi: 10.3390/ijerph192214921 (PMC9690722; doi:10.3390/ijerph192214921)

Supplementary Material

Supplementary Table S1: Search strategies.

**Database**	**Scopus**<br>**Date: 14/12/2021**		**Results**
**Search Strategy**	#1	TITLE-ABS-KEY ("pressure ulcer*")	14328
	#2	TITLE-ABS-KEY ("decubitus ulcer*")	4980
	#3	TITLE-ABS-KEY ("pressure sore*")	4127
	#4	TITLE-ABS-KEY (bedsore*)	1011
	#5	TITLE-ABS-KEY ("bed sore*")	421
	#6	#1 OR #2 OR #3 OR #4 OR #5	20733
	#7	TITLE-ABS-KEY ("olive oil")	28122
	#8	#6 AND #7	16

**Database**	**PubMed**<br>**Date: 14/12/2021**		**Results**
**Search Strategy**	#1	Pressure Ulcer[Mesh]	13
	#2	Pressure Ulcer*[tiab]	8,323
	#3	Decubitus Ulcer*[tiab]	2,033
	#4	Pressure Sore*[tiab]	3,189
	#5	Bedsore*[tiab]	580
	#6	Bed Sore*[tiab]	239
	#7	#1 OR #2 OR #3 OR #4 OR #5 OR #6	17,536
	#8	Olive Oil[Mesh]	5,464
	#9	Olive Oil[tiab]	11,53
	#10	#8 OR #9	12,379
	#11	#7 AND #10	10

**Database**	**EMBase (OVID)**<br>**Date: 14/12/2021**		**Results**
**Search Strategy**	#1	exp decubitus/	24825
	#2	(Pressure adj5 Ulcer*).ti,ab.	11568
	#3	(Decubitus adj5 Ulcer*).ti,ab.	2656
	#4	(Pressure adj5 Sore*).ti,ab.	4221
	#5	Bedsore*.ti,ab.	934
	#6	(Bed adj5 Sore*).ti,ab.	499
	#7	or/1-6	29170

	#8	exp olive oil/	15580
	#9	(Olive adj3 Oil).ti,ab.	15735
	#10	8 or 9	19798
	#11	7 and 10	19

**Database**	**Cochrane Library (Wiley)**<br>**Date: 14/12/2021**		**Results**
**Search Strategy**	#1	MeSH descriptor: [Pressure Ulcer] explode all trees	795
	#2	(Pressure NEAR/5 Ulcer*):ti,ab,kw	1791
	#3	(Decubitus NEAR/5 Ulcer*):ti,ab,kw	187
	#4	(Pressure NEAR/5 Sore*):ti,ab,kw	579
	#5	Bedsore*:ti,ab,kw	118
	#6	(Bed NEAR/5 Sore*):ti,ab,kw	92
	#7	#1 OR #2 OR #3 OR #4 OR #5 OR #6	2306
	#8	MeSH descriptor: [Olive Oil] explode all trees	576
	#9	(Olive NEAR/3 Oil*):ti,ab,kw	2216
	#10	#8 OR #9	2216
	#11	#7 AND #10	21

**Database**	**CINAHL (Complete-EBSCO)**<br>**Date: 14/12/2021**		**Results**
**Search Strategy**	S1	(MH "Pressure Ulcer+")	15,154
	S2	TI (Pressure N5 Ulcer*) OR AB (Pressure N5 Ulcer*)	9,579
	S3	TI (Decubitus N5 Ulcer*) OR AB (Decubitus N5 Ulcer*)	526
	S4	TI (Pressure N5 Sore*) OR AB (Pressure N5 Sore*)	1,967
	S5	TI (Bed N5 Sore*) OR AB (Bed N5 Sore*)	124
	S6	TI Bedsore* OR AB Bedsore*	230
	S7	S1 OR S2 OR S3 OR S4 OR S5 OR S6	17,862
	S8	(MH "Olive Oil")	1,234
	S9	TI (Olive N3 Oil*) OR AB (Olive N3 Oil*)	2,295
	S10	S8 OR S9	2,811
	S11	S7 AND S10	15

**Database**	**Web of Science (Core Collection)**<br>**Date: 14/12/2021**		**Results**
**Search Strategy**	#1	TS=Pressure Ulcer*	14,785

	#2	TI=Pressure Ulcer* OR AB=Pressure Ulcer*	10,925
	#3	TI=Decubitus Ulcer* OR AB=Decubitus Ulcer*	994
	#4	TI=Pressure Sore* OR AB=Pressure Sore*	3,110
	#5	TI=Bedsore* OR AB=Bedsore*	307
	#6	TI=Bed Sore* OR AB=Bed Sore*	332
	#7	#1 OR #2 OR #3 OR #4 OR #5 OR #6	18,368
	#8	TS=Olive Oil	27,431
	#9	TI=Olive Oil OR AB=Olive Oil	20,798
	#10	#8 OR #9	27,431
	#11	#10 AND #7	20

**Database**	**Global Health (OVID)**<br>**Date: 14/12/2021**		**Results**
**Search Strategy**	#1	(Pressure adj5 Ulcer*).ti,ab.	554
	#2	(Decubitus adj5 Ulcer*).ti,ab.	162
	#3	(Pressure adj5 Sore*).ti,ab.	191
	#4	Bedsore*.ti,ab.	91
	#5	(Bed adj5 Sore*).ti,ab.	63
	#6	or/1-5	984
	#7	(Olive adj3 Oil*).ti,ab.	11376
	#8	6 and 7	1

**Database**	**LILACS (BVS-Eng)**<br>**Date: 14/12/2021**		**Results**
**Search Strategy**	#1	**(MH Pressure Ulcer OR Ulcer$ OR Bedsore$ OR Escara$ OR Bed-Sore$) AND (MH Olive Oil OR ((Olive OR Oliva) AND (Aceite$ OR Oil$ OR Azeite$))) [Words]**	2

Supplementary Table S2: List of records identified in databases after removing duplicates.

1. Treatment Update. McKnight's Long-Term Care News 2015; 36: 9-9.

2. Treatment Update. McKnight's Long-Term Care News 2016; 37: 8-8.

3. Adedapo AA, Jimoh FO, Koduru S, et al. Assessment of the medicinal potentials of the methanol extracts of the leaves and stems of Buddleja saligna. Bmc Complementary and Alternative Medicine 2009; 9. DOI: 10.1186/1472-6882-9-21.

4. Costa AMA, Donnato-Trancoso A and Romana-Souza B. Olive oil administration improves cutaneous wound healing of pressure ulcers in mice. FASEB Journal 2016; 30.

5. Díaz-Valenzuela A, García-Fernández FP, Carmona Fernández P, et al. Effectiveness and safety of olive oil preparation for topical use in pressure ulcer prevention: Multicentre, controlled, randomised, and double-blinded clinical trial. Int Wound J 2019; 16: 1314-1322. 2019/09/03. DOI: 10.1111/iwj.13191.

6. Díaz-Valenzuela A, Valle Cañete MJ, Carmona Fernández PJ, et al. Eficacia en la prevención de úlceras por presión del aceite de oliva virgen extra frente a los ácidos grasos hiperoxigenados: resultados intermedios de un estudio de no inferioridad. Gerokomos 2014; 25: 74-80. DOI: 10.4321/s1134-928x2014000200005.

7. Dilly RdS, Barretta C, Matos CH, et al. Nutritional status and consumption of inflammatory and anti-inflammatory foods by patients with inflammatory bowel diseases^ien Estado nutricional e consumo de alimentos inflamatórios e anti-inflamatórios por pacientes portadores de doenças inflamatórias intestinais^ipt. J coloproctol (Rio J, Impr) 2020; 40: 99-104.

8. Donato-Trancoso A, Monte-Alto-Costa A and Romana-Souza B. Olive oil-induced reduction of oxidative damage and inflammation promotes wound healing of pressure ulcers in mice. J Dermatol Sci 2016; 83: 60-69. 2016/04/20. DOI: 10.1016/j.jdermsci.2016.03.012

10.1016/j.jdermsci.2016.03.012. Epub 2016 Apr 1.

9. Franco MDL, Valenzuela SC and Miranda JMG. Efficacy of extra-virgin olive oil versus hyperoxygenated fatty acids for pressure ulcers prevention: A systematic review with meta-analysis. Gerokomos 2016; 27: 117-122.

10. Gefen A and Ousey K. Update to device-related pressure ulcers: SECURE prevention. COVID-19, face masks and skin damage. Journal of Wound Care 2020; 29: 245-259. DOI: http://dx.doi.org/10.12968/jowc.2020.29.5.245.

11. Hekmatpou D, Rahzani K, Mehrabi F, et al. The effect of aloe vera clinical trials on prevention and healing of skin wound: A systematic review. Iranian Journal of Medical Sciences 2019; 44: 1-9.

12. Hong LZ. Radix linderae essential oil improving the immunity activities and preventing the occurrence of decubitus in aged people. Journal of Medicinal Plants Research 2011; 5: 3733-3738.

13. Irct2013111014634N. Effect of topical Olive oil on prevention of bedsore in intensive care unit patients. https://trialsearchwhoint/Trial2aspx?TrialID=IRCT2013111014634N2 2014.

14. Irct2015102724410N. Olive & Bed sore. https://trialsearchwhoint/Trial2aspx?TrialID=IRCT2015102724410N2 2015.

15. Irct20150519022320N. Comparative investigating of the Effect of Aloe Vera gel and Olive Oil on Incidence of pressure ulcer in patients Hospitalized. https://trialsearchwhoint/Trial2aspx?TrialID=IRCT20150519022320N20 2019.

16. Irct20160914029817N. Investigating the effect of Olive and Bitter almonds on pressure ulcers. https://trialsearchwhoint/Trial2aspx?TrialID=IRCT20160914029817N8 2018.

17. Irct20170124032147N. The Effect of Olive Oil and Sweet Almonds on the prevention of Bed Sore. https://trialsearchwhoint/Trial2aspx?TrialID=IRCT20170124032147N4 2018.

18. Irct20180227038885N. The comparison of fish oil, olive oil and honey of bed sore. https://trialsearchwhoint/Trial2aspx?TrialID=IRCT20180227038885N1 2018.

19. Irct20181105041563N. Comparative evaluation of the effect of Aloe Vera Gel, Olive oil and Compound Aloe Vera Gel- Olive oil on Prevention of pressure ulcer. https://trialsearchwhoint/Trial2aspx?TrialID=IRCT20181105041563N4 2019.

20. Irct20181228042154N. exercise-induced muscle damage; prophylactic and therapeutic countermeasures. https://trialsearchwhoint/Trial2aspx?TrialID=IRCT20181228042154N1 2019.

21. Irct20200105046008N. The effect of Aleo Vera and Olive Oil preventive dressing usage on pressure injury development. https://trialsearchwhoint/Trial2aspx?TrialID=IRCT20200105046008N1 2020.

22. Jara CP, Mendes NF, Prado TPD, et al. Bioactive Fatty Acids in the Resolution of Chronic Inflammation in Skin Wounds. Advances in Wound Care 2020; 9: 472-490. DOI: http://dx.doi.org/10.1089/wound.2019.1105.

23. Karimi Z, Mousavizadeh A, Rafiei H, et al. The Effect of Using Olive Oil and Fish Oil Prophylactic Dressings on Heel Pressure Injury Development in Critically Ill Patients. Clinical Cosmetic and Investigational Dermatology 2020; 13: 59-65. DOI: 10.2147/ccid.S237728.

24. Krowchuk HV and Woodgate R. TOWARD EVIDENCE-BASED PRACTICE. MCN: The American Journal of Maternal Child Nursing 2014; 39: 336-338. DOI: 10.1097/NMC.0000000000000072.

25. López Franco MD, Chiquero Valenzuela S and Garrido Miranda JM. Eficacia del aceite de oliva virgen extra frente a los ácidos grasos hiperoxigenados en la prevención de las úlceras por presión: revisión sistemática con metaanálisis. Gerokomos 2016; 27: 117-122.

26. Lupiáñez-Pérez I, Morilla-Herrera JC, Ginel-Mendoza L, et al. Effectiveness of olive oil for the prevention of pressure ulcers caused in immobilized patients within the scope of primary health care: study protocol for a randomized controlled trial. Trials 2013; 14: 348. 2013/10/25. DOI: 10.1186/1745-6215-14-348

10.1186/1745-6215-14-348.

27. Lupiañez-Pérez I, Morilla-Herrera JC, Kaknani-Uttumchandani S, et al. A cost minimization analysis of olive oil vs. hyperoxygenated fatty acid treatment for the prevention of pressure ulcers in primary healthcare: A randomized controlled trial. Wound Repair Regen 2017; 25: 846-851. 2017/09/19. DOI: 10.1111/wrr.12586

10.1111/wrr.12586. Epub 2017 Nov 6.

28. Lupiañez-Perez I, Uttumchandani SK, Morilla-Herrera JC, et al. Topical olive oil is not inferior to hyperoxygenated fatty aids to prevent pressure ulcers in high-risk immobilised patients in home care. Results of a multicentre randomised triple-blind controlled non-inferiority trial. PLoS One 2015; 10: e0122238. 2015/04/18. DOI: 10.1371/journal.pone.0122238

10.1371/journal.pone.0122238. eCollection 2015.

29. Miraj S, Pourafzali S, Ahmadabadi Z, et al. Effect of olive oil in preventing the development of pressure ulcer grade one in intensive care unit patients. Int J Prev Med 2020; 11. Article. DOI: 10.4103/ijpvm.IJPVM_545_18.

30. Moore ZE and Webster J. Dressings and topical agents for preventing pressure ulcers. Cochrane Database Syst Rev 2018; 12: Cd009362. 2018/12/12. DOI: 10.1002/14651858.CD009362.pub3

10.1002/14651858.CD009362.pub3.

31. Nct. Olive Oil's Cream Effectiveness in Prevention of Pressure Ulcers in Immobilized Patients in Primary Care. https://clinicaltrialsgov/show/NCT01595347 2012.

32. Orhan B and Manav G. Effects of ozone oil on wound healing in rats with pressure ulcer. Oxid Commun 2018; 41: 329-335. Article.

33. Panahi Y, Izadi M, Sayyadi N, et al. Comparative trial of Aloe vera/olive oil combination cream versus phenytoin cream in the treatment of chronic wounds. J Wound Care 2015; 24: 459-460, 462-455. 2015/10/22. DOI: 10.12968/jowc.2015.24.10.459

10.12968/jowc.2015.24.10.459.

34. Poursadra E, Anvari-Tafti M, Dehghani A, et al. Comparing the Effect of Henna Oil and Olive Oil on Pressure Ulcer Grade One in Intensive Care Units Patients. Adv Biomed Res 2019; 8: 68. 2020/01/04. DOI: 10.4103/abr.abr_207_19

10.4103/abr.abr_207_19. eCollection 2019.

35. Raza Meer MQ. Soft tissue technique and olive oil massage prevent pressure sores in central pontine mylenosis (CPM). Rawal Medical Journal 2014; 39: 228-229.

36. Saeedinejad S, Rostaminejad A, Abdi N, et al. Olive Oil as Treatment for Bedsore: a Randomized Clinical Trial. International Journal of Advanced Biotechnology and Research 2017; 8: 1172-1176.

37. Sakazaki F, Kataoka H, Okuno T, et al. Ozonated olive oil enhances the growth of granulation tissue in a mouse model of pressure ulcer. Ozone-Science & Engineering 2007; 29: 503-507. DOI: 10.1080/01919510701618205.

38. Schanuel FS, Saguie BO and Monte-Alto-Costa A. Olive oil promotes wound healing of mice pressure injuries through NOS-2 and Nrf2. Applied Physiology, Nutrition & Metabolism 2019; 44: 1199-1208. DOI: 10.1139/apnm-2018-0845.

39. Sönmez M and Yapucu Güneş Ü. Preventive effect of extra virgin olive oil on pressure injury development: A randomized controlled trial in Turkey. Complementary Therapies in Clinical Practice 2020; 40: N.PAG-N.PAG. DOI: 10.1016/j.ctcp.2020.101208.

40. Taheri M and Amiri-Farahani L. Anti-Inflammatory and Restorative Effects of Olives in Topical Application. Dermatology Research and Practice 2021; 2021: 9927976. DOI: http://dx.doi.org/10.1155/2021/9927976.

41. Tiderencel KA and Brody RA. Evidence-Based Interventions for Diabetic Heel Ulcers: Nutrition-Focused Management of a Rehabilitation Patient. Topics in Clinical Nutrition 2017; 32: 305-315. DOI: 10.1097/TIN.0000000000000116.

42. Vahabzadeh D, Valizadeh Hasanloei MA and Vahdat Shariatpanahi Z. Effect of high-fat, low-carbohydrate enteral formula versus standard enteral formula in hyperglycemic critically ill patients: a randomized clinical trial. International Journal of Diabetes in Developing Countries 2019; 39: 173-180. DOI: http://dx.doi.org/10.1007/s13410-018-0660-z.

43. Vitsos A, Tsagarousianos C, Vergos O, et al. Efficacy of a Ceratothoa oestroides Olive Oil Extract in Patients With Chronic Ulcers: A Pilot Study. Int J Low Extrem Wounds 2019; 18: 309-316. 2019/06/27. DOI: 10.1177/1534734619856143

10.1177/1534734619856143. Epub 2019 Jun 25.

44. Wadhwani GG. Topical Homeopathic Application in a Stage IV Decubitus Ulcer: A Homeopathic Clinical Snapshot. American Journal of Homeopathic Medicine 2018; 111: 23-25.

45. Zulim LFdC, Nai GA, Giuffrida R, et al. Comparison of the efficacy of 0. 03% tacrolimus eye drops diluted in olive oil and linseed oil for the treatment of keratoconjunctivitis sicca in dogs^ien Comparação do colírio tacrolimus 0, 03% em óleo de oliva e linhaça no tratamento da ceratoconjuntivite seca em cães^ipt. Arq bras oftalmol 2018; 81: 293-301.

Supplementary Table S3: Excluded records in title and abstract review.

Article	Reason
Assessment of the medicinal potentials of the methanol extracts of the leaves and stems of Buddleja saligna	No olive oil
Anti-Inflammatory and Restorative Effects of Olives in Topical Application	No prevention of pressure ulcers
Bioactive Fatty Acids in the Resolution of Chronic Inflammation in Skin Wounds	No prevention of pressure ulcers
Comparative evaluation of the effect of Aloe Vera Gel, Olive oil and Compound Aloe Vera Gel- Olive oil on Prevention of pressure ulcer	Clinical trial registration
Comparative investigating of the Effect of Aloe Vera gel and Olive Oil on Incidence of pressure ulcer in patients Hospitalized	Clinical trial registration
Comparative trial of Aloe vera/olive oil combination cream versus phenytoin cream in the treatment of chronic wounds	No prevention of pressure ulcers
Comparison of the efficacy of 0. 03% tacrolimus eye drops diluted in olive oil and linseed oil for the treatment of keratoconjunctivitis sicca in dogs^ien Comparação do colírio tacrolimus 0, 03% em óleo de oliva e linhaça no tratamento da ceratoconjuntivite seca em cães^ipt	No prevention of pressure ulcers
Comparing the Effect of Henna Oil and Olive Oil on Pressure Ulcer Grade One in Intensive Care Units Patients	Patients with pressure ulcer
Dressings and topical agents for preventing pressure ulcers	No randomized controlled trial
Effects of ozone oil on wound healing in rats with pressure ulcer	Non-human study
Effectiveness of olive oil for the prevention of pressure ulcers caused in immobilized patients within the scope of primary health care: study protocol for a randomized controlled trial	No randomized controlled trial
Effect of high-fat, low-carbohydrate enteral formula versus standard enteral formula in hyperglycemic critically ill patients: a randomized clinical trial	No olive oil
Effect of topical Olive oil on prevention of bedsore in intensive care unit patients	Clinical trial registration

Efficacy of a Ceratothoa oestroides Olive Oil Extract in Patients With Chronic Ulcers: A Pilot Study	Patients with pressure ulcer
Efficacy of extra-virgin olive oil versus hyperoxygenated fatty acids for pressure ulcers prevention: A systematic review with meta-analysis	No randomized controlled trial
Eficacia del aceite de oliva virgen extra frente a los ácidos grasos hiperoxigenados en la prevención de las úlceras por presión: revisión sistemática con metaanálisis	No randomized controlled trial
Evidence-Based Interventions for Diabetic Heel Ulcers: Nutrition-Focused Management of a Rehabilitation Patient	No prevention of pressure ulcers
exercise-induced muscle damage; prophylactic and therapeutic countermeasures	No olive oil
Investigating the effect of Olive and Bitter almonds on pressure ulcers	Clinical trial registration
Nutritional status and consumption of inflammatory and anti-inflammatory foods by patients with inflammatory bowel diseases^ien Estado nutricional e consumo de alimentos inflamatórios e anti-inflamatórios por pacientes portadores de doenças inflamatórias intestinais^ipt	No olive oil
Olive & Bed sore	Clinical trial registration
Olive Oil's Cream Effectiveness in Prevention of Pressure Ulcers in Immobilized Patients in Primary Care	Clinical trial registration
Olive oil administration improves cutaneous wound healing of pressure ulcers in mice	Non-human study
Olive oil-induced reduction of oxidative damage and inflammation promotes wound healing of pressure ulcers in mice	Non-human study
Olive oil promotes wound healing of mice pressure injuries through NOS-2 and Nrf2	Non-human study
Ozonated olive oil enhances the growth of granulation tissue in a mouse model of pressure ulcer	Non-human study
Radix linderae essential oil improving the immunity activities and preventing the occurrence of decubitus in aged people	No olive oil

Soft tissue technique and olive oil massage prevent pressure sores in central pontine mylenosis (CPM)	No prevention of pressure ulcers
The comparison of fish oil, olive oil and honey of bed sore	Clinical trial registration
The effect of Aleo Vera and Olive Oil preventive dressing usage on pressure injury development	Clinical trial registration
The effect of aloe vera clinical trials on prevention and healing of skin wound: A systematic review	No randomized controlled trial
The Effect of Olive Oil and Sweet Almonds on the prevention of Bed Sore	Clinical trial registration
Topical Homeopathic Application in a Stage IV Decubitus Ulcer: A Homeopathic Clinical Snapshot	No prevention of pressure ulcers
Toward Evidence-based practice	No randomized controlled trial
Treatment Update	No randomized controlled trial
Treatment Update	No randomized controlled trial
Update to device-related pressure ulcers: SECURE prevention. COVID-19, face masks and skin damage	No olive oil

Supplementary Table S4: Excluded studies in full-text review.

N	Study	Reason
1	Eficacia en la prevención de úlceras por presión del aceite de oliva virgen extra frente a los ácidos grasos hiperoxigenados: resultados intermedios de un estudio de no inferioridad	Preliminary results
2	A cost minimization analysis of olive oil vs. hyperoxygenated fatty acid treatment for the prevention of pressure ulcers in primary healthcare: A randomized controlled trial: Prevention of pressure ulcers in primary healthcare	Preliminary results
3	Effect of Olive Oil in Preventing the Development of Pressure Ulcer Grade One in Intensive Care Unit Patients	Wrong population (the study was performed in patients with ulcers)
4	The Effect of Using Olive Oil and Fish Oil Prophylactic Dressings on Heel Pressure Injury Development in Critically Ill Patients	No randomized controlled trial

Supplementary Table S5: The PRISMA Statement checklist[1].

Section/topic	#	Checklist item	Reported on page #
**TITLE**			
Title	1	Identify the report as a systematic review, meta-analysis, or both.	1
**ABSTRACT**			
Structured summary	2	Provide a structured summary including, as applicable: background; objectives; data sources; study eligibility criteria, participants, and interventions; study appraisal and synthesis methods; results; limitations; conclusions and implications of key findings; systematic review registration number.	1
**INTRODUCTION**			
Rationale	3	Describe the rationale for the review in the context of what is already known.	1-2
Objectives	4	Provide an explicit statement of questions being addressed with reference to participants, interventions, comparisons, outcomes, and study design (PICOS).	2
**METHODS**			
Protocol and registration	5	Indicate if a review protocol exists, if and where it can be accessed (e.g., Web address), and, if available, provide registration information including registration number.	2
Eligibility criteria	6	Specify study characteristics (e.g., PICOS, length of follow-up) and report characteristics (e.g., years considered, language, publication status) used as criteria for eligibility, giving rationale.	2
Information sources	7	Describe all information sources (e.g., databases with dates of coverage, contact with study authors to identify additional studies) in the search and date last searched.	2
Search	8	Present full electronic search strategy for at least one database, including any limits used, such that it could be repeated.	2
Study selection	9	State the process for selecting studies (i.e., screening, eligibility, included in systematic review, and, if applicable, included in the meta-analysis).	2-3
Data collection process	10	Describe method of data extraction from reports (e.g., piloted forms, independently, in duplicate) and any processes for obtaining and confirming data from	3

		investigators.	
Data items	11	List and define all variables for which data were sought (e.g., PICOS, funding sources) and any assumptions and simplifications made.	3
Risk of bias in individual studies	12	Describe methods used for assessing risk of bias of individual studies (including specification of whether this was done at the study or outcome level), and how this information is to be used in any data synthesis.	3
Summary measures	13	State the principal summary measures (e.g., risk ratio, difference in means).	3-4
Synthesis of results	14	Describe the methods of handling data and combining results of studies, if done, including measures of consistency (e.g., I^2) for each meta-analysis.	3-4
Risk of bias across studies	15	Specify any assessment of risk of bias that may affect the cumulative evidence (e.g., publication bias, selective reporting within studies).	3-4
Additional analyses	16	Describe methods of additional analyses (e.g., sensitivity or subgroup analyses, meta-regression), if done, indicating which were pre-specified.	4
**RESULTS**			
Study selection	17	Give numbers of studies screened, assessed for eligibility, and included in the review, with reasons for exclusions at each stage, ideally with a flow diagram.	4
Study characteristics	18	For each study, present characteristics for which data were extracted (e.g., study size, PICOS, follow-up period) and provide the citations.	5-6
Risk of bias within studies	19	Present data on risk of bias of each study and, if available, any outcome level assessment (see item 12).	6
Results of individual studies	20	For all outcomes considered (benefits or harms), present, for each study: (a) simple summary data for each intervention group (b) effect estimates and confidence intervals, ideally with a forest plot.	7-8
Synthesis of results	21	Present results of each meta-analysis done, including confidence intervals and measures of consistency.	7-8
Risk of bias across studies	22	Present results of any assessment of risk of bias across studies (see Item 15).	6
Additional analysis	23	Give results of additional analyses, if done (e.g., sensitivity or subgroup analyses, meta-regression [see Item 16]).	7
**DISCUSSION**			

Summary of evidence	24	Summarize the main findings including the strength of evidence for each main outcome; consider their relevance to key groups (e.g., healthcare providers, users, and policy makers).	9
Limitations	25	Discuss limitations at study and outcome level (e.g., risk of bias), and at review-level (e.g., incomplete retrieval of identified research, reporting bias).	9
Conclusions	26	Provide a general interpretation of the results in the context of other evidence, and implications for future research.	10
**FUNDING**			
Funding	27	Describe sources of funding for the systematic review and other support (e.g., supply of data); role of funders for the systematic review.	10

[1] Moher D, Liberati A, Tetzlaff J, Altman DG, The PRISMA Group (2009). Preferred Reporting Items for Systematic Reviews and Meta-Analyses: The PRISMA Statement. PLoS Med 6(6): e1000097. doi:10.1371/journal.pmed1000097

Supplementary Figure S1: Subgroup analysis in patients in ICU (forest plot).

Study	Experimental Events	Experimental Total	Control Events	Control Total	Weight (common)	Weight (random)	Risk Ratio IV, Fixed + Random, 95% CI
Sonmez	11	65	21	64	67.8%	67.8%	0.52 [0.27; 0.98]
Saeedinejad	5	35	12	35	32.2%	32.2%	0.42 [0.16; 1.06]
**Total (fixed effect, 95% CI)**		**100**		**99**	**100.0%**	**--**	**0.48 [0.28; 0.82]**
**Total (random effects, 95% CI)**					**--**	**100.0%**	**0.48 [0.28; 0.82]**

Heterogeneity: Tau^2 = 0; Chi^2 = 0.14, df = 1 (P = 0.71); I^2 = 0%

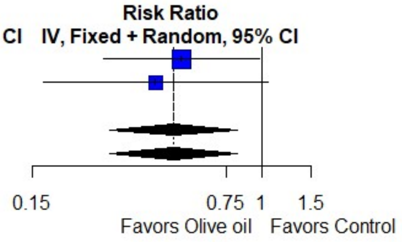

Supplementary Figure S2: Subgroup analysis in studies with HOFA as control (forest plot).

Study	Experimental Events	Experimental Total	Control Events	Control Total	Weight (common)	Weight (random)	Risk Ratio IV, Fixed + Random, 95% CI
Díaz-Valenzuela	11	283	18	288	42.5%	42.5%	0.62 [0.30; 1.29]
Lupiañez-Perez	16	314	21	260	57.5%	57.5%	0.63 [0.34; 1.18]
**Total (fixed effect, 95% CI)**		**597**		**548**	**100.0%**	**--**	**0.63 [0.39; 1.01]**
**Total (random effects, 95% CI)**					**--**	**100.0%**	**0.63 [0.39; 1.01]**

Heterogeneity: Tau^2 = 0; Chi^2 = 0.00, df = 1 (P = 0.98); I^2 = 0%

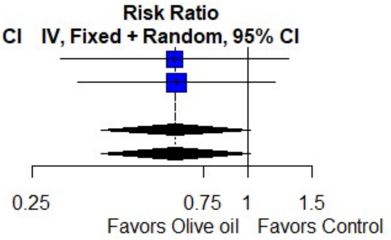

Supplement: Supplementary file 1 [file ijerph-19-14921-s001.zip › Supplementary Materials_Tables & Figures.pdf]
