# Supplementary material for: Efficacy and Safety of Topical Application of Olive Oil for Preventing Pressure Ulcers: A Systematic Review and Meta-Analysis of Randomized Controlled Trials
Source: Int J Environ Res Public Health. 2022 Nov 13;19(22):14921. doi: 10.3390/ijerph192214921 (PMC9690722; doi:10.3390/ijerph192214921)
Supplement: Supplementary file 1 [file ijerph-19-14921-s001.zip › Supplementary Material_File S1.pdf]

Supplementary Material S1. Analysis codes.

```
# Supplementary Material S1 Analysis codes

###
Title:	"Efficacy and safety of topical application of olive oil for
preventing pressure ulcers: A systematic review and meta-analysis of randomized
controlled trials"
Authors:	"Akram Hernández-Vásquez, ahernandez@usil.edu.pe"
Date: "31/03/2022"
###

### R version
"All analyses included in this meta-analysis were conducted using R version 4.0.3"

### Required packages
#If required install the following packages
install.packages("tibble", repos="http://cran.r-project.org")
install.packages("metafor", repos="http://cran.r-project.org")
install.packages("meta", repos="http://cran.r-project.org")
install.packages("robvis", repos="http://cran.r-project.org")

# Load packages (after installed) with the library function
library(tibble)
library(metafor)
library(meta)
library(robvis)

### Citations for included packages
citation("tibble")
citation("metafor")
citation("meta")

#
# Load data for Meta-analysis: Efficacy
#
data_eficacia <- tibble(
  author = c("Díaz-Valenzuela","Lupiañez-Perez","Sonmez","Saeedinejad"),
  year = c(2019,2015,2020,2017),
  event.e = c(11,16,11,5),
  n.e = c(283,314,65,35),
  event.c = c(18,21,21,12),
  n.c = c(288,260,64,35),
)
```

```
View(data_eficacia)

#
# Load data for Meta-analysis: Safety
#
data_seguridad <- tibble(
   author = c("Díaz-Valenzuela","Lupiañez-Perez","Sonmez"),
   year = c(2019,2015,2020),
   event.e = c(0,1,0),
   n.e = c(283,314,65),
   event.c = c(1,2,0),
   n.c = c(288,260,64),
)

View(data_seguridad)

##################################################################################################
# Meta-analysis from Meta Package: Binary outcome 2x2
##################################################################################################
### Model 1: Efficacy
m1 <- metabin(event.e, n.e, event.c, n.c,
                    data = data_eficacia, sm = "RR",
                    studlab = author, method = "Inverse")
summary(m1)
funnel(m1)
forest(m1, xlim = c(0.15, 1.5), layout = "RevMan", label.right="Favors Control", label.left="Favors Olive oil")
metainf(m1)
forest(metainf(m1))
metainf(m1, pooled="random")
forest(metainf(m1, pooled="random"))
forest(metainf(m1, pooled="random"), xlim = c(0.3, 1.5), layout = "RevMan", label.right="Favors Control", label.left="Favors Olive oil")

### Subgroup HOFA
m1S1 <- metabin(event.e, n.e, event.c, n.c,
                    data = data_eficacia, sm = "RR",
                    studlab = author, method = "Inverse", subset = c(1, 2))
summary(m1S1)
funnel(m1S1)
forest(m1S1, xlim = c(0.25, 1.5), layout = "RevMan", label.right="Favors Control", label.left="Favors Olive oil")
metainf(m1S1)
forest(metainf(m1S1))
metainf(m1S1, pooled="random")
forest(metainf(m1S1, pooled="random"))
forest(metainf(m1S1, pooled="random"), xlim = c(0.25, 1.5), layout = "RevMan", label.right="Favors Control", label.left="Favors Olive oil")
```

```r
### Subgroup
m1S2 <- metabin(event.e, n.e, event.c, n.c,
                        data = data_eficacia, sm = "RR",
                        studlab = author, method = "Inverse", subset = c(3, 4))
summary(m1S2)
funnel(m1S2)
forest(m1S2, xlim = c(0.15, 1.5), layout = "RevMan", label.right="Favors Control", label.left="Favors Olive oil")
metainf(m1S2)
forest(metainf(m1S2))
metainf(m1S2, pooled="random")
forest(metainf(m1S2, pooled="random"))
forest(metainf(m1S2, pooled="random"), xlim = c(0.15, 1.5), layout = "RevMan", label.right="Favors Control", label.left="Favors Olive oil")

### Model 2: Safety
m2 <- metabin(event.e, n.e, event.c, n.c,
                     data = data_seguridad, sm = "RR",
                     studlab = author, method = "Inverse")
summary(m2)
funnel(m2)
forest(m2, xlim = c(0.01, 8.5), layout = "RevMan", label.right="Favors Control", label.left="Favors Olive oil")
metainf(m2)
forest(metainf(m2))
metainf(m2, pooled="random")
forest(metainf(m2, pooled="random"), xlim = c(0.01, 8.5))
forest(metainf(m2, pooled="random"), xlim = c(0.01, 8.5), layout = "RevMan", label.right="Favors Control", label.left="Favors Olive oil")


##################################################################################################
# Plots of risk-of-bias assessments
##################################################################################################

#
# Load data of RoB 2
#

data_rob2 <- tibble(
   Study = c("Díaz-Valenzuela","Lupiañez-Perez","Sonmez","Saeedinejad"),
   D1 = c("Low", "Low", "Low", "Low"),
   D2 = c("Low", "Low", "Low", "Low"),
   D3 = c("Low", "Low", "Low", "High"),
   D4 = c("Low", "Low", "Low", "Low"),
   D5 = c("Low", "Low", "Low", "Low"),
   Overall = c("Low", "Low", "Low", "Low"),
```

```
   Weight = c(571, 574, 129, 70),
)

View(data_rob2)

##Plots
rob_summary(data_rob2, tool = "ROB2", overall = TRUE)
rob_traffic_light(data_rob2, tool = "ROB2")
```
